# Identification of quantitative trait loci underlying five major agronomic traits of soybean in three biparental populations by specific length amplified fragment sequencing (SLAF-seq)

**DOI:** 10.7717/peerj.12416

**Published:** 2021-12-14

**Authors:** Bo Hu, Yuqiu Li, Hongyan Wu, Hong Zhai, Kun Xu, Yi Gao, Jinlong Zhu, Yuzhuo Li, Zhengjun Xia

**Affiliations:** 1 Key Laboratory of Soybean Molecular Design Breeding, Northeast Institute of Geography and Agroecology, The Innovative Academy of Seed Design, Chinese Academy of Sciences, Harbin, China; 2 University of Chinese Academy of Sciences, Beijing, China

**Keywords:** Soybean, QTL, SLAF-seq, Genetic map, Flowering time

## Abstract

Flowering time, plant height, branch number, node numbers of main stem and pods per plant are important agronomic traits related to photoperiodic sensitivity, plant type and yield of soybean, which are controlled by multiple genes or quantitative trait loci (QTL). The main purpose of this study is to identify new QTL for five major agronomic traits, especially for flowering time. Three biparental populations were developed by crossing cultivars from northern and central China. Specific loci amplified fragment sequencing (SLAF-seq) was used to construct linkage map and QTL mapping was carried out. A total of 10 QTL for flowering time were identified in three populations, some of which were related to *E1* and *E2* genes or the other reported QTL listed in Soybase. In the Y159 population (Xudou No.9 × Kenfeng No.16), QTL for flowering time on chromosome 4, *qFT4_1* and *qFT4_2* were new. Compared with the QTL reported in Soybase, 1 QTL for plant height (PH), 3 QTL for branch number (BR), 5 QTL for node numbers of main stem, and 3 QTL for pods per plant were new QTL. Major *E* genes were frequently detected in different populations indicating that major the *E* loci had a great effect on flowering time and adaptation of soybean. Therefore, in order to further clone minor genes or QTL, it may be of great significance to carefully select the genotypes of known loci. These results may lay a foundation for fine mapping and clone of QTL/genes related to plant-type, provided a basis for high yield breeding of soybean.

## Introduction

Soybean is an important commercial crop in the world, and one of the major sources of vegetable protein and oil (Wilcox, 2004). However, due to the photoperiodic sensitivity of soybean, a given superior cultivar can only be planted in a specific ecological area or geographical latitude, thus limiting its potential for widespread cultivation. The growth period of soybean, namely flowering time and maturity, is an important ecological index of photoperiod phenomenon (Panthee et al., 2007), it is also closely related to yield, quality and planting area of soybean.

Up to now, a total of 473 QTL associated with flowering time and maturation of soybean have been preserved in public Soybase. More than ten genes related to flowering time and maturity have been cloned; The most important of these are *E* series genes. In 2008, *E4* gene was cloned and identified as phytochrome A (*GmphyA2*) (Liu et al., 2008). The *E3* gene was cloned in 2009 by map-based cloning, and *E3* was found to be another copy of the phytochrome A (*GmphyA3*) (Watanabe et al., 2009). When the *E3* and *E4* loci carry dominant alleles, the flowering time and maturation of soybeans were both delayed, while when the genotype was homozygous recessive, the flowering time and maturation of soybean were both accelerated. *E2* gene was a homologous gene of *GIGANTEA* (*GI*) in Arabidopsis; there were a few alleles of *E2* in soybean varieties. Under natural light, the *e2* genotype may lead to early flowering by up-regulating the expression abundance of *GmFT2a*. *E2* gene contributed significantly to the growth period of soybean, but had little effect on the photoperiod response (Watanabe et al., 2011). *E1* can inhibit flowering and delay soybean growth period. Xia successfully cloned *E1* gene using Harosoy near-isogenic line, and discovered that the *E1* gene is a unique transcription factor of legume crops (Xia et al., 2012). Flowering and maturity of soybean are controlled by major genes, and four key maturity loci (*E1–E4*) are helpful to understand the mechanism of flowering and maturity. Allelic combinations of these genes determine the diversity of maturation and their adaptation to different latitudes (Li et al., 2017; Jiang et al., 2014). In recent years, some genes that can regulate flowering, such as *E9* (Kong et al., 2014), *J* (Lu et al., 2017), *FT* (Kong et al., 2010; Zhai et al., 2014a) and *PRR* (Li et al., 2019) have been isolated.

Phenotypes, such as plant height, branch number, node numbers of main stem and pods per plant of soybean, are important agronomic traits, it is closely related to planting density, lodging resistance, canopy structure and photosynthetic efficiency of soybean, and has an important effect on the soybean yield. In Soybase database, QTL for plant height, branch number, node numbers of main stem and pods per plant are 230, 21, 38 and 51, respectively. Plant height, branch number and nodes are important plant architecture traits, and there are many studies on genetic and QTL mapping in soybean (Liu et al., 2013; Panthee et al., 2007; Zhang et al., 2004; He et al., 2014; Sayama et al., 2010; Assefa et al., 2019). Pods per plant are an especially important factor in determining soybean yield (Board & Tan, 1995). QTL for flowering and pods number were identified using 152 recombinant inbred lines and a linkage map of 306 markers. Two main QTL (*qpn-Chr11* and *qpn-Chr20*) were detected for pods number (Zhang et al., 2010).

Quantitative traits are mostly regulated by multiple genes and affected by environment, and QTL mapping requires the construction of high-quality genetic linkage map. Polymorphic molecular markers are needed to construct the genetic map. High throughput of molecular markers development is very necessary. With the rapid development of sequencing technology, molecular markers have been transformed from traditional RFLP, RAPD and AFLP to the SSR or SNP markers. These molecular markers have significantly improved in speed, cost, and amount of information, speeding up the construction of genetic maps, gene mapping, and cloning. Specific loci amplified fragment sequencing (SLAF-seq) is a set of simplified genome sequencing technology, which has the advantages of long reads, high flux, flexible design, etc. Up to 100,000 labels can be developed at a time to obtain genome-wide cariation information (Sun et al., 2013). It has been widely used in the genetic map construction, QTL mapping, gene location and molecular breeding. Some important QTL were identified in soybean using this technique. A high-density genetic map covering a 2,144.85 cM soybean genome was constructed using 3,255 SLAF-markers. Six QTL for plant height and eleven QTL for flowering time were mapped (Ca et al., 2017). The map consisted of 5,785 SLAFs on 20 linkage groups (LGs) with a genome size of 2,255.18 cM. The 20 linkage groups had high collinearity with the reference genome (Li et al., 2014 ).

In the previous study, 180 cultivars were genotyped at the *E1* to *E4* loci, and the flowering time and maturity of these cultivars were investigated; Statistical analysis showed that allelic variations at each of four loci had a significant effect on flowering time and maturity (Zhai et al., 2014b). A total of 30 consistent QTNs were detected for flowering time (R1) and maturity (R7 and R8) on 16 chromosomes by genome-wide association study (GWAS) of 235 cultivars, and the known *E* loci were detected in different environment and year (Wang et al., 2018). Previous results have also showed that in the same genotypic populations, the time range to flowering and maturation is quite large, suggesting that there is some unknown genetic factor.

Therefore, one of the objectives of this study is to identify new or minor QTL for flowering time. Plant height, branch number, node numbers of main stem and pods per plant are important plant-type and yield traits, QTL mapping of these traits can lay a foundation for further cloning related genes, which is helpful to breed high-yield soybean cultivars.

## Materials and Methods

### Plant materials and field trails

The parents for developing biparental populations were selected according to different genetic background and soybean variety origin (Zhai et al., 2014b). Three populations were developed from a cross of Daheiqi and Pixian Ruantiaozhi (named Y32); Liaodou No.15 and Jilin No.35 (named Y133); Xudou No.9 and Kenfeng No.16 (named Y159), respectively. Randomized interval field trials were performed for each population. In 2015, three F_2_ genetic populations were planted using a consistent sowing method in the experimental field in Harbin. The row spacing was 60 cm, the line length was 5 m, and the plant spacing was 20 cm. In order to maintain relative uniform condition and obtain exact data in field, the emergence data of each individuals was recorded, and the plant spacing was consistent. Conventional field management were adopted, individual plant harvested and major agronomic traits were investigated.

### Phenotyping

The investigation standards was based on soybean germplasm resources description specification and data standard and other research articles published (Fehr et al., 1971; Sayama et al., 2010 and Chen et al., 2007). Flowering time (FT) is the number of days from the emergence (VE) to the opening of the first flower (R1) found at any node on the main stem. After the soybean matured, each plant was harvested into a mesh bag to keep the plant intact. Plant height (PH) is the length from the cotyledonary node to the top of the plant. Branch number (BR) indicates the effective branches of the main stem. Node numbers of main stem (Nodes) indicate the number of nodes from the cotyledonary node to the top of the main stem. Pods per plant (Pods) indicate the number of pods with normal seeds.

### Genotyping and construction of genetic linkage map

DNA was extracted from leaves by hexadecyltrimethylammonium bromide (CTAB) method with slight modification (Murray & Thompson, 1980; Xia et al., 2007).

To develop high-density molecular markers, 104 individuals of Y32 population, 105 individuals of Y133 population, and 104 individuals of Y159 population were randomly selected from the F_2_ population, and genotyping was performed by SLAF-seq technology in Biomarker Technologies Corporation. Firstly, in order to select the most suitable scheme and to predict the reference genome by enzyme digestion, the enzyme *Hae*III was selected in this study. The length of restriction fragments between 264 bp and 364 bp was defined as the SLAF tag. The sample DNA was digested with enzyme according to the program to obtain SLAF tag and treated with 3′end plus A. Sequencing connectors, PCR amplification, purification, mixing and gel cutting were connected to the target fragment, and the prepared library was sequenced with Illumina Hiseq 2500.

Reads of each sample were obtained by identifying the raw data with the Dual-index connector sequence. The reads with connector sequence and N content more than 10% of the total length were filtered. Reads were compared with the genome to search for the polymorphisms SLAF tags. To ensure the quality of genetic map, polymorphisms SLAF tags were filtered. The filtering principle was that the depth of parental sequence was less than 10X, the number of SNPs was more than three, and the markers were severe deviation (*P* < 0.05). The modified LOD (MLOD) between the two tags was calculated to further filter the tags whose MLOD was less than three (Stam, 1993). High-density genetic map including 20 linkage groups (LGs) was constructed using High Map software (Liu et al., 2014). The genetic linkage map was constructed by interval plotting, and the recombination rate was converted into cell distance units (cM) by Kosambi function (*P* = 0.001).

### QTL mapping and epistatic analysis by ICIM

After the genetic linkage map was constructed, the QTL for the main agronomic traits, such as flowering time, plant height, branch number, node numbers of main stem and pods per plant, were mapped by QTL Icimapping_4.0 software with the phenotypic data (Li, Ye & Wang, 2007). Composite interval mapping (ICIM-ADD) was used for QTL mapping, and LOD = 2.5 was the minimum threshold for QTL existence. Compared to the reported QTL loci or genes in Soybase, QTL with physical distance less than 5 Mb from known QTL were listed. After dividing into sub-populations, the sampe size with *E1* and *e1* background were 36 and 29 in Y32 population, respectively. The sample size with *E1* and *e1* background were 32 and 30 in Y133 population, respectively. The sample with *E2* background was 34 in Y159 population. QTL mapping was also conducted in the sub-populations.

Epistatic analysis of QTL was conducted by ICIM-EPI method. LOD = 5 was the minimum threshold for QTL with epistatic interaction.

### Genotyping of the *E1* and *E2* genes

Genotyping of the *E1* and *E2* loci was performed in populations according to published procedures, respectively (Xu et al., 2013; Zhai et al., 2014b). Due to the sample problem, several individuals of three populations were excluded for verification.

The *E1* was genotyped by amplifying genomic DNA with the primer pair (Forward: TCAGATGAAAGGGAGCAGTGTCAAAAGAAGT; Reverse: TCCGATCTCATCACCTTTCC). The PCR was performed using the following program: 30 cycles at 94 °C for 20 s, 58 °C for 30 s, and 72 °C for 30 s. A 443/444 bp fragment was amplified and used to distinguish *e1-as* from *E1* with the aid of relevant restriction enzymes, *Taq*I. The 142 bp fragment was amplified with primers (Forward: GAAGCCCATCAGAGCATGTCTTATT; Reverse: GAGGCAGAGCCAAAGCCTAT) and the *Dra*I digestion was performed. The PCR procedurewas the same as that of *E1* amplification. The fragment amplified from *E2* allele was not cut off; while that from *e2* allele could be cut into 115 bp and 27 bp fragments.

## Results

### Phenotypic variations in the F_2_ populations

**Figure 1:**
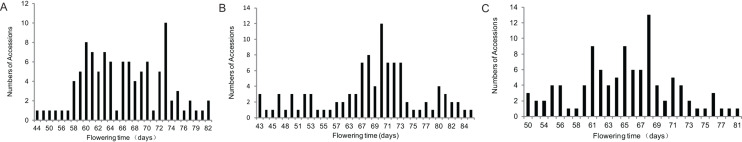
Frequency distributions of flowering time in three F_2_ populations.

Based on previous study, soybean cultivars with different genetic background of the major *E* genes in northern China and Huang-Huai-Hai region were selected for population development. The parents of the three populations shared the same genotypes at *E3* and *E4* loci (Zhai et al., 2014b); however, the genotypes of *E1* or *E2* loci were different (Table 1). Flowering time of the three populations showed major separations (Table 2, Fig. 1). Plant height, branch number, node numbers of main stem and pods per plant were also statistically analyzed and showed in Table 2 in detail. All of the traits showed a large separation, and genetic variation. The absolute values of skewness and kurtosis of each trait were less than one and the deviation was small, indicating that these traits were in line with normal distribution, and the map construction and QTL analysis could be carried out.

**Table 1:** Details of soybean materials used in this study.

Population name	Parents	Genotype of *E1–E4*	Origin	Number of individuals	FT^1^	PH^2^	BR^3^	Nodes^4^	Pods^5^
Y32	Daheiqi	*e1-ase2E3HaE4*	Liaoning Province, China	144	51 ± 1.8	87 ± 1.1	2 ± 1.0	21 ± 1.1	110 ± 1.7
	Pixian Ruantiaozhi	*E1E2E3HaE4*	Jiangsu Province, China		94 ± 1.4	98 ± 1.5	4 ± 1.4	23 ± 1.3	121 ± 1.5
Y133	Liaodou 15	*E1e2E3HaE4*	Liaoning Province, China	137	68 ± 2.0	79 ± 1.3	4 ± 1.2	24 ± 1.0	109 ± 1.6
	Jilin 35	*e1-ase2E3HaE4*	Jilin Province, China		53 ± 1.9	99 ± 1.8	2 ± 1.2	19 ± 1.5	118 ± 1.9
Y159	Xudou 9	*E1E2E3HaE4*	Jiangsu Province, China	140	87 ± 1.3	60 ± 1.7	3 ± 1.6	20 ± 1.6	115 ± 1.8
	Kenfeng 16	*E1e2E3HaE4*	Heilongjiang Province, China		54 ± 1.6	85 ± 1.2	1 ± 1.0	19 ± 1.3	124 ± 1.9

**Notes:**

1 Flowering time (FT), unit: days.

2 Plant height (PH), unit: centimeters.

3 Branch number (BR).

4 Node numbers of main stem (Nodes).

5 Pods per plant (Pods).

**Table 2:** Variations in phenotypic characteristics for three F_2_ populations.

	Trait name^1^	Mean^2^	StdError^3^	Skewness^4^	Kurtosis^5^	Minimum^6^	Maximum^7^	W-test^8^
Y32	FT	66.00	6.97	−0.19	0.36	44.00	82.00	0.97
	PH	111.64	24.50	−0.68	1.42	32.00	170.00	0.95
	BR	6.13	2.03	0.21	−0.3	2.00	11.00	0.96
	Node	23.28	15.91	8.94	84.69	9.00	24.00	0.31
	Pod	146.09	64.36	0.70	1.47	13.00	373.00	0.96
Y133	FT	66.74	10.63	−0.62	−0.31	43.00	88.00	0.91
	PH	99.90	25.91	−0.32	−0.25	34.00	160.00	0.97
	BR	4.51	1.94	0.47	0.07	0.00	10.00	0.95
	Node	19.20	3.91	−0.47	−0.41	8.00	26.00	0.95
	Pod	119.97	49.60	0.92	2.31	16.00	315.00	0.95
Y159	FT	64.78	6.49	−0.23	−0.06	50.00	81.00	0.97
	PH	89.89	7.09	−0.19	0.03	68.00	106.00	0.99
	BR	4.14	1.94	0.31	−0.32	0.00	9.00	0.95
	Node	21.10	2.89	−1.31	2.69	9.00	26.00	0.90
	Pod	121.45	47.83	0.67	0.89	33.00	306.00	0.97

**Notes:**

1 Flowering time (FT)(unit: days), Plant height (PH)(unit: centimeters), Branch number (BR), Node numbers of main stem (Nodes), Pods per plant (Pods).

2 Mean of the phenotypic trait.

3 Standard deviation of the phenotypic trait.

4 Skewness of the phenotypic trait.

5 Kurtosis of the phenotypic trait.

6 Minimum value of the phenotypic trait.

7 Maximum value of the phenotypic trait.

8 The Shapiro Wilk W-statistic for the test of normality.

### Marker genotyping and genetic map construction

In order to rapidly develop molecular markers for genetic linkage mapping, three F_2_ populations were genotyped by SLAF-seq. The Q30 of sequencing data was all above 80% and the GC content was around 40% after removing the low quality markers (Table S1).

**Figure 2:**
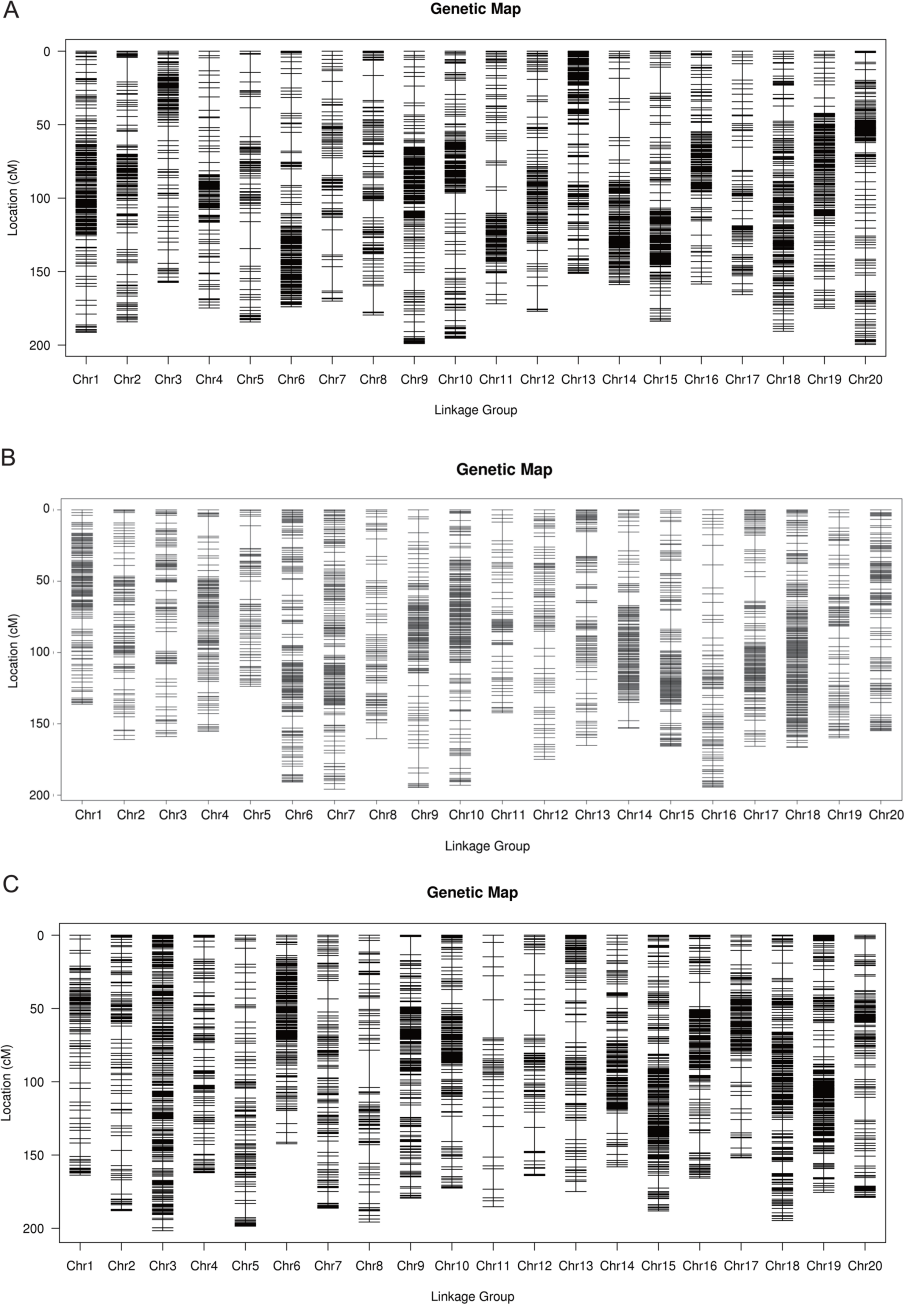
The genetic linkage maps of three F_2_ populations (A–C).

The total number of SLAF markers and polymorphism markers of the three populations were showed in Table S2. In Y32 population, the total length of the genetic map was 3,542.26 cM and the average distance was 0.68 cM (Table 3). In Y133 population, the markers using for constructing linkage map was 6,629, the total length of genetic map of 6,629 cM and the average distance of 0.50 cM (Table 3). In the Y159 population, 6,069 markers were obtained after filtering. The total length of genetic linkage map was 3,527.43 cM and the average distance being 0.58 cM (Table 3). The constructed high-density genetic maps were shown in Fig. 2.

**Table 3:** Description of characteristics of linkage groups in three F_2_ populations.

Population	Total marker	Total distance (cM)	Average distance (cM)	Max gap (cM)	Gap < 5 cM
Y32	5,248	3,542.26	0.68	19.66	0.96
Y133	6,629	3,312.97	0.50	17.24	0.97
Y159	6,069	3,527.43	0.58	26.01	0.95

### QTL mapping for main agronomic traits by ICIM

On the basis of the high-density genetic linkage map, QTL IciMapping 4.0 software and ICIM-ADD model were used to map the important agronomic traits such as flowering time, plant height, branch number, node numbers of main stem and pods per plant.

**Figure 3:**
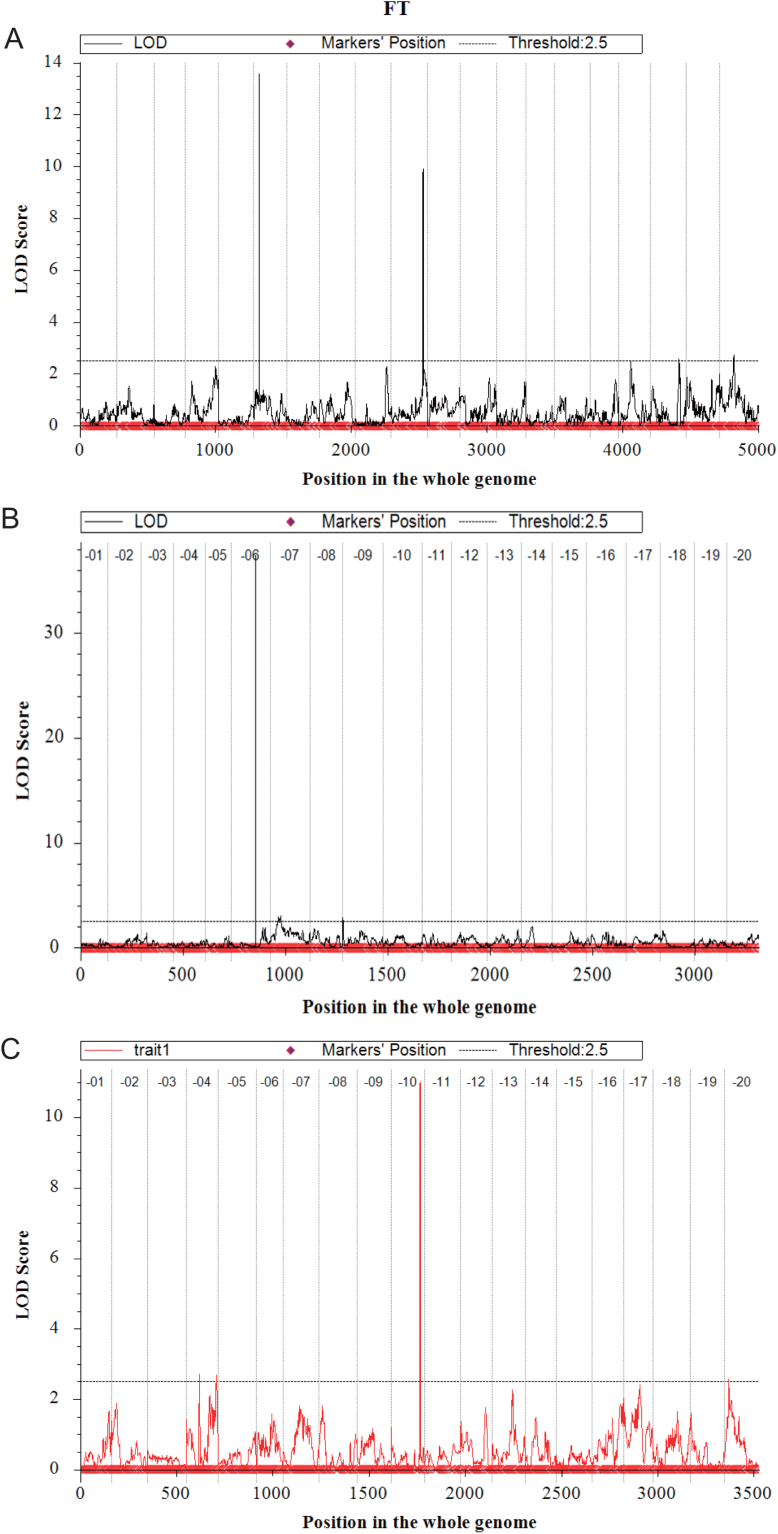
Quantitative trait loci for flowering time mapping by ICIM-ADD in three F_2_ populations (A–C).

In the Y32 population, the QTL for flowering time were located on chromosome 6, 10, 18 and 20 (Fig. 3). The LOD value and PVE of QTL on chromosome 6 and chromosome 10 were 13.57, 32.30% and 9.90, 21.59%, respectively, higher than those on other chromosomes. Three QTL for plant height were detected on chromosome 7 and 15, with PVE of 12.49%, 10.75% and 14.22%, respectively. Only one QTL for branch number on chromosome 8. Three QTL for total pods number were detected on chromosome 1, 7 and 9 (Table 4).

**Table 4:** Details of the QTL detected by ICIM method in three F_2_ populations.

	QTL^1^	Chr^2^	LeftMarker^3^	Physical position (bp)	RightMarker^4^	Physical position (bp)	LOD^5^	PVE (%)^6^	Add^7^	Dom^8^	Distance to known QTL or gene (kb)	QTL in SoyBase or known gene
Y32	*qFT6_1*	6	Marker323324	23537158	Marker358360	27338462	13.57	32.30	1.19	−9.10	3,329.22	*E1* (Xia et al., 2012)
	*qFT10_1*	10	Marker776629	45046804	Marker863420	45440450	9.90	21.59	1.60	−7.72	Included	*E2* (Watanabe et al., 2011)
	*qFT18_1*	18	Marker3233026	7595593	Marker3272581	5563090	2.57	4.94	−0.10	4.18	1,387.98	First flower 9-2 (Tasma et al., 2001)
	*qFT20_1*	20	Marker1141036	39449778	Marker1208177	39743944	2.70	4.53	1.53	−2.43	2,607.41	First flower 25-3 (Kuroda et al., 2013)
	*qPH7_1*	7	Marker3543364	36305294	Marker3558465	37867013	2.66	12.49	−2.50	20.10	Inside	Plant height 37-5 (Yao et al., 2015)
	*qPH15_1*	15	Marker2248389	6350243	Marker2232956	6291315	2.99	10.75	−0.91	−22.41	1,187.28	Plant height 29-4 (Liu et al., 2011)
	*qPH15_2*	15	Marker2240384	47919789	Marker2283233	47849933	3.87	14.22	11.21	−4.93	3,101.93	Plant height 13-3 (Specht et al., 2001)
	*qBR8_1*	8	Marker489330	10849470	Marker425892	11184428	2.55	10.67	0.85	−0.12		
	*qPod1_1*	1	Marker2932559	52563296	Marker2849051	51269516	2.60	11.25	19.28	26.23		
	*qPod7_1*	7	Marker3636272	39034339	Marker3559742	43637244	2.56	14.14	−15.44	−43.88	1,954.73	Pod number 8-2 (Kuroda et al., 2013)
	*qPod9_1*	9	Marker574929	39196944	Marker585751	39126794	2.99	13.19	−4.94	55.40	Inside	Pod number 4-2 (Vieira et al., 2006)
Y133	*qFT6_1*	6	Marker1511272	22576376	Marker1477657	25662576	37.41	79.84	−13.44	3.41	2,368.44	*E1* (Xia et al., 2012)
	*qFT9_1*	9	Marker3130054	20109767	Marker3059482	19768764	2.85	2.55	−2.24	−0.36	Inside	First flower 24-2 (Kuroda et al., 2013)
	*qPH16_1*	16	Marker2161238	156191	Marker2199160	175385	2.65	5.80	−3.13	−10.17	547.79	Plant height 13-5 (Specht et al., 2001)
	*qPH19_1*	19	Marker1629638	44862177	Marker1741286	44948002	8.46	22.18	14.85	8.80	85.6	Plant height 10-4 (Orf et al., 1999)
	*qBR10_1*	10	Marker2018083	42634098	Marker1984857	42592504	2.61	8.55	0.73	−0.71	Inside	Branching 2-1 (Li et al., 2008)
	*qBR16_1*	16	Marker2202176	4335021	Marker2227376	4061146	2.82	10.65	0.26	1.33		
	*qBR16_2*	16	Marker2194053	32878039	Marker2180126	32881100	2.70	8.88	−0.69	−0.81		
	*qNode2_1*	2	Marker1045753	13457001	Marker1043109	14241316	3.56	5.13	0.97	1.24		
	*qNode6_1*	6	Marker1591836	29389703	Marker1422918	20262849	17.54	38.63	−3.56	1.20	43.96	Node number 2-2 (Zhang et al., 2004)
	*qNode16_1*	16	Marker2199160	175187	Marker2153289	119447	4.03	7.02	−0.35	−1.99		
	*qNode19_1*	19	Marker1603266	44854038	Marker1666593	45184768	10.94	23.75	2.43	1.66		
	*qPod19_1*	19	Marker1661937	1188581	Marker1609122	527556	2.52	13.48	5.22	−31.45		
Y159	*qFT4_1*	4	Marker2190645	8278635	Marker2198514	14131214	2.70	7.13	−2.49	0.25		
	*qFT4_2*	4	Marker2247000	49076978	Marker2157183	49623244	2.67	6.26	0.96	2.94		
	*qFT10_1*	10	Marker2556622	44734722	Marker2594984	44920804	11.01	35.33	−6.31	1.04	373.93	*E2* (Watanabe et al., 2011)
	*qFT20_1*	20	Marker548749	1452527	Marker564445	2408622	2.57	6.06	−1.68	2.52	1,494.79	First flower 20-3 (Funatsuki et al., 2005)
	*qPH12_1*	12	Marker160225	34446165	Marker286957	36996073	2.93	12.00	3.21	1.25	Included	Plant height 17-12 (Kabelka et al., 2004)
	*qPH14_1*	14	Marker3540947	12499348	Marker3510677	13522667	2.58	8.45	−2.54	2.17		
	*qPH18_1*	18	Marker3020509	5811373	Marker2865777	5814304	3.23	11.86	−3.40	0.65	Inside	Plant height 23-6 (Reinprecht et al., 2006)
	*qNode7_1*	7	Marker344604	2461951	Marker385437	4606180	2.64	9.60	1.39	0.15		
	*qNode12_1*	12	Marker160225	34446165	Marker286957	36996073	4.45	16.90	1.60	0.18		
	*qPod13_1*	13	Marker965589	33594593	Marker929333	34347994	2.77	11.07	−14.97	22.20	4,860.44	Pod number 12-1 (Qi et al., 2014)
	*qPod20_1*	20	Marker527124	1593395	Marker479294	1533077	2.51	9.78	−22.42	−4.02		

**Notes:**

1 The nomenclature of the QTL included three parts: name, trait and chromosome name.

2 Chr, chromosome.

3 The left markers flanking the likelihood of odds (LOD)-value peak interval for the QTL.

4 The right markers flanking the likelihood of odds (LOD)-value peak interval for the QTL.

5 LOD score calculated from single marker analysis.

6 Proportion of phenotypic variance explained (PVE) by a QTL.

7 The estimated additive effect of the alleles of the maternal parent.

8 Estimated dominance effect of the marker.

In the Y133 population, the QTL for flowering time was located on chromosome 6 and 9 (Fig. 3), and the major QTL was on chromosome 6, with LOD value of 37.41, and PVE of 79.84%. Two QTL for plant height were located on chromosome 19, with LOD value of 8.46 and PVE of 22.18%. In addition, three QTL for branch number on chromosome 10 and 16 were detected. Four QTL for node numbers of main stem were identified, in which the LOD and PVE of *qNode6_1* and *qNode19_1* were 17.54, 38.63% and 10.94, 23.75%, respectively. One QTL for pods per plant was located on chromosome 19 (Table 4).

Four QTL for flowering time were located on chromosome 4, 10 and 20, respectively in Y159 (Fig. 3). Among them, the QTL located on chromosome 10 was the major QTL, with LOD value of 11.01 and PVE of 35.33%. Three QTL regulating plant height were detected on chromosome 12, 14 and 18, respectively. Two QTL for node numbers of main stem were located on chromosome 7 and 12, respectively. The LOD of *qNode12_1* was 4.45, and the PVE was 16.90%. Two QTL for pods per plant were located on chromosome 13 and 20, respectively (Table 4).

### QTL analysis

In three populations, a total of 34 QTL were identified for five traits. The corresponding QTL or known genes reported in Soybase were listed in Table 4. The genotypes of *E1* and *E2* of parents are shown in Fig. S3. Both in Y32 and Y133 populations, a QTL for flowering time with high PVE (32.30%, 79.84%) near the known *E1* loci was detected. A QTL for flowering time near the *E2* loci on chromosome 10 was identified in Y32 and Y159 populations, with PVE of 21.59% and 35.33%, respectively. Two QTL for the flowering time on Chr04 in Y159 population were new QTL, and there were two genes (*Glyma.04G093900* (*AGL24*), *Glyma.04G101500* (*CRY1*)) related to flowering in the interval of *qFT4_1* in Y159 population.

**Figure 4:**
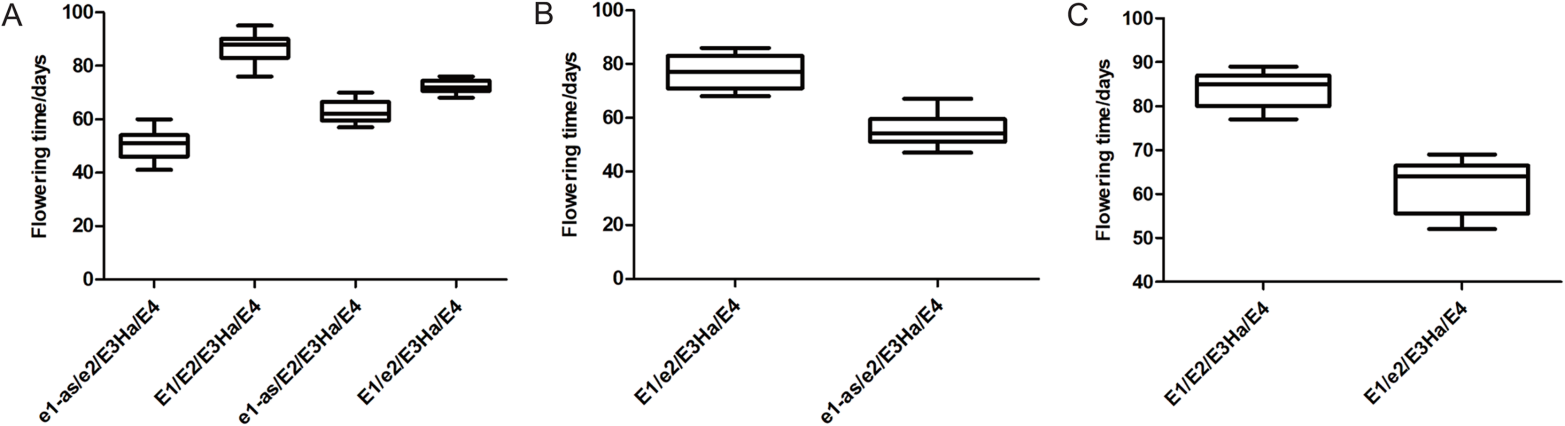
Flowering time of individuals with different genotypes in three populations, respectively. X-axis: different genotype combination of E1–E4, Y-axis: flowering time of each combination. ((A) Y32 population, (B) Y133 population, and (C) Y159 population).

Flowering time was affected by major *E* genes and different allelic combination. The *E1* and *E2* genotypes of each individual were identified in three populations, and the flowering time of the individuals with the same genotype was statistically analyzed (Fig. 4). The flowering time of the individuals with *E1E2E3HaE4* genotype was late, and the individuals with *e1-ase2E3HaE4* genotype was earlier than the other genotype. *E1* gene has a greater effect on flowering time than *E2*. The flowering time of the individuals having the same genotype was not exactly same in three populations, indicating that *E1* and *E2* played different roles in different genetic backgrounds, or the other genes regulating flowering time were at play.

Plant height, branch number, node numbers of main stem and pods per plant were important agronomic traits affecting plant architecture and yield of soybean. A total of 8 QTL for plant height in three populations were detected, and seven of them were corresponding to the reported QTL in Soybase. The QTL, *qPH14_1*, in Y159 population was new QTL. There were four QTL for branch number identified in Y32 and Y133 populations, of which three were new loci with short interval. Six QTL for node numbers of main stem were detected in Y133 and Y159 population, the QTL *qNode6_1* in Y133 population was near the reported loci with about 43.96 kb, and the other five QTL were new loci. Six QTL for pods per plant were detected in three populations, *qPod1_1* of Y32, *qPod19_1* of Y133 and *qPod20_1* of Y159 were three new QTL (Table 4).

*E1* and *E2* are the main genetic factors controlling flowering time, maturity and geographic adaption in Chinese cultivars. Therefore, it is difficult to detect other QTL with small effects due to the large influence of major genes on flowering time. Based on different *E1* or *E2* alleles, each population was divided into two sub-populations, and QTL for five agronomic traits with high LOD and PVE were identified in the sub-populations (Tables S3–S5).

### Analysis of QTL epistatic effect by ICIM-EPI method

**Figure 5:**
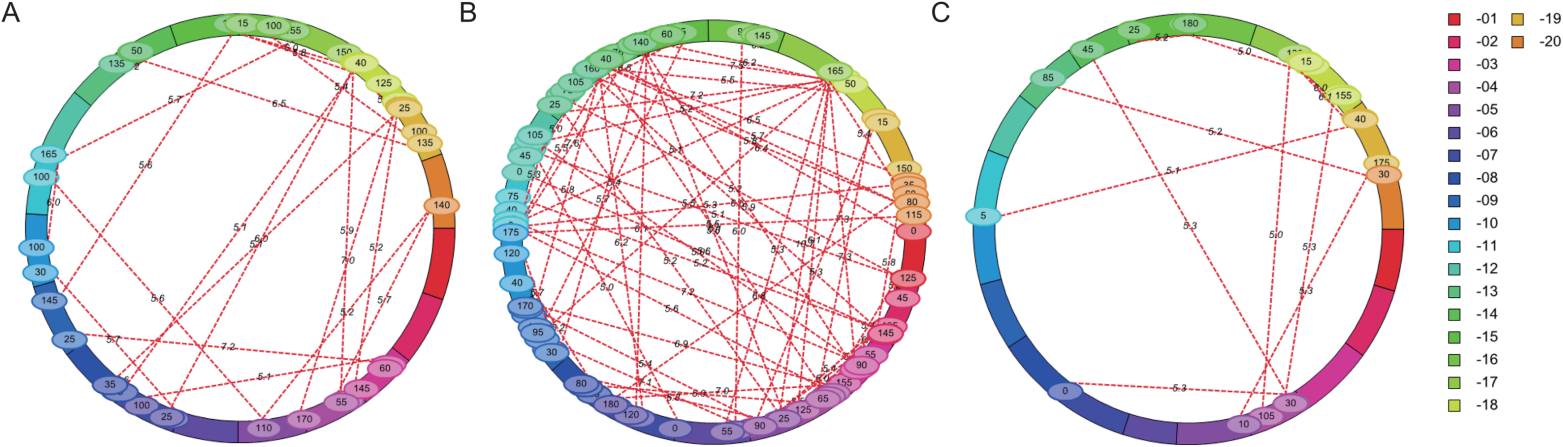
Epistatic interacting QTL of flowering time in three populations detected by ICIM-EPI ((A) Y32 population, (B) Y133 population, and (C) Y159 population).

The flowering time of soybean is a complex quantitative trait regulated by many genes or QTL loci. Epistasis is the main genetic basis of complex traits. In this study, epistatic effect of QTL for flowering time was analyzed by ICIM-EPI. In Y32, Y133 and Y159 populations, 22, 64, and 11 pairs of epistatic QTL were detected, respectively (Fig. 5). The PVE value of the epistatic QTL in Y133 population was around 1.5%, and that of the epistatic QTL in Y159 population was higher (around 6.06%).

## Discussion

Soybean is an important oil and commercial crop, which not only provides plant protein and oil for human beings, but also serves as one of the main ingredients of bean pulp, which is necessary for human dietary pattern and animal husbandry. Flowering time, plant height, branch number, node numbers of main stem and nodes per plant are the important agronomic traits that affect soybean yield, and they are all quantitative traits regulated by multiple genes. QTL mapping and genetic analysis of these traits can reveal the genetic characteristics of each trait, provide the basis for marker-assisted breeding, and available locus for molecular design breeding.

High-throughput sequencing is helpful for QTL/gene mapping and functional research. The construction of high density genetic linkage map is a necessary condition for QTL mapping, and the development of polymorphic molecular markers is a prerequisite for the construction of linkage map. As a new generation of molecular marker, SNP has been widely used for its advantages of high density, good stability and easy automatic analysis. SLAF-seq is a high-resolution strategy for single nucleotide polymorphism genotyping that has been developed in recent years. SLAF-seq has many advantages, such as longer reads, high throughput and flexible scheme design, etc. SLAF-seq can develop 100,000 labels at a time to obtain genome-wide variation information. It has been widely applied in genetic map construction and QTL mapping, gene location and molecular breeding. The final constructed map contained 5,308 markers distributed on 20 linkage groups with a length of 2,655.68 cM (Qi et al., 2014).

The map length was 2,909.46 cM, and the mean distance between markers was 0.57 cM. A total of 35 QTL related to plant height, 100-seeds weight, oil content and protein content were found (Zhang et al., 2018). SLAF-seq was used to construct a genetic map of the recombinant inbred lines (Luheidou 2 × Nanhuizao, F_5:8_), and the QTL for isoflavone content across various environments were identified with this map (Li et al., 2014). In this study, SLAF-seq technology was used to genotype three F_2_ generation populations to construct genetic linkage maps. After quality control, 5,248, 6,629 and 6,069 markers were developed in Y32, Y133 and Y159 populations, with total length of 3,542.26, 3,312.97 and 3,527.43 cM, respectively. Collinearity analysis was carried out based on the position of the markers on the genome and the genetic map. The results showed that most of the markers sequences on each linkage group were consistent with the genome, implying that the map had good collinearity and high accuracy. The map constructed with extensive and high-density markers developed by SLAF-seq was of high quantity.

At present, there are few strategies for QTL/gene mapping based on natural population or hybrid populations. Genome-Wide Association Studies (GWAS) is a widely used method for gene location. Population structure becomes complex due to the need to collect a large number of experimental materials. Meanwhile, this method can hardly detect rare variants. Compared with GWAS, gene/QTL mapping based on hybrid population has certain advantages and plays an irreplaceable role. The genetic background of this population is pure; there is relatively little variation between parents. As a temporary population, the F_2_ generation population has the advantages of relatively simple and time-saving construction, and contains rich genetic information. However, F_2_ population also have some limitations or disadvantages. Phenotypic data of F_2_ population are poor in reliability and repeatability, and it is hard to detect minor genes. Therefore, it is necessary to evaluate phenotypic data before QTL mapping. In this study, the phenotypic data of three F_2_ hybrid populations were investigated in detail, and the results showed that there was a large separation of traits and genetic variation. The absolute values of skewness and kurtosis of each trait were less than one or the deviation is small, which conforms to the normal distribution and meets the requirements of QTL mapping. Due to the limitations of F_2_ population, it is necessary to identify reconbinants and construct larger subgroups to further fine mapping the QTL.

Soybean cultivars can be grown across the world in a wide range of latitudes from 50°N to 35°S (Watanabe, Harada & Abe, 2012). This is mainly attributed to the rich genetic variability and different allelic combinations of genes or loci that influence the flowering time and maturity. *E1*, *E2*, *E3*, *E4* have different impacts on maturity and their allelic variation and combination determine the diversification of soybean maturity and adaptation to different latitudes. Jiang et al. (2014) found that the more recessive alleles at *E* genes, the earlier the cultivars matured. In the US, a molecular model for soybean maturity based on the alleles underlying the major maturity loci: *E1*, *E2* and *E3*, can significantly improve soybean breeding efficiency. Among the 48 Chinese cultivars, the *E1* and *e2* allele were predominant, and the results also showed that China generally had the most variation of *E1* and *E2* alleles as well as *E* genotype groups (Langewisch et al., 2017). A total of 59 cultivars sensitive to photoperiod were selected from different ecologies. Among them, *E1*/*e2*/*E3*/*E4* was more abundant in the range of N 18°–N 42°, while *E1*/*E2*/*E3*/*E4* was distributed south of N 39° (Jiang et al., 2014). Based on preliminary study of the group, Zhai et al. (2014b) evaluated 180 cultivars from six sites for 2 years of flowering time and maturity, and classified them into *E1*-*E4* genotypes. The cultivars with genotypes of *e1-as*, *e2*, and *E3* and *E4* are mainly from Jilin and Liaoning provinces. A total of 67 cultivars or accessions with recessive *e2*, *E1*, *E3* and *E4* were the largest one of eight groups; the geographic distribution of this group was much diversified, from the northern Heilongjiang province, to southern Jiangsu province (the region along the Yangtze River). *E1*/*E2*/*E3*/*E4* group were geographically from the southern areas, Jiangsu, Shanghai and Anhui provinces. Similarly, in the previous study, a total of 30QTNs related to flowering time and maturity of soybean were identified by GWAS of 235 cultivars from different countries using medium-density SNP sequences, most of which corresponded to known *E1* to *E4* genes or the reported QTL region in Soybase (Wang et al., 2018). In this study, parents of three populations were selected from the soybean cultivars among the 235 cultivars using for GWAS analysis. The parents from the Northeast China and Huang-Huai-Hai region were carried with various alleles of *E1* or *E2* genes, and the difference of these two *E* loci contributed to different adaptation. A total of 10 QTL for flowering time were detected, some of which were also corresponding to the known *E1* and *E2* genes, which further explained the core functions of *E1* and *E2* in different regions affecting flowering time and maturity. Similar to the reported QTL for flowering time, some QTL corresponding to *E1* or *E2* were frequently detected, indicating that they had the great effect on flowering time.

Due to the dominant role of major *E* genes in flowering and maturity, the genotypes of known gene loci should be considered to eliminate the effect of major *E* genes. In order to further clone minor genes or QTL, it is better to select the parents with the same alleles on the major gene loci. However, some studies had mapped the *E1* or *E2* QTL locus and found new QTL after dividing a population into two sub-populations with the same allele (Lu et al., 2016; Wang et al., 2019; Yang et al., 2017). In this study, each individual of the population was genotyped according to the *E1* or *E2* loci, and a population was divided into two sub-populations with different genotypes at *E1* or *E2* loci, and genetic map construction and QTL mapping were performed again. Some additional QTL with high PVE were detected; it might be an effective strategy for detecting minor QTL in the presence of major QTL, while in order to further fine mapping the candidate gene, it is necessary to expand the population size and find important recombinants. The flowering time of soybean is a complex network regulated by multiple genes. There are still new flowering genes or QTL loci that need to be detected. The interaction between flowering genes needs to be further studied, which lays a foundation for revealing the flowering regulatory network in soybean.

The QTL for important quality traits (protein content, oil content), yield traits (pods per plant, 100-seed weight), plant height, branch number, node numbers of main stem had also been identified and studied (Zhang et al., 2010; Yin et al., 2018; Chen et al., 2007; Li et al., 2008). In Soybase database, some reported QTL for these traits were listed in detail. In this study, plant height, branch number, node numbers of main stem and pods per plant were also investigated, and QTL mapping was also carried out. A total of eight plant height related QTL were identified in three populations. Compared with the QTL reported in Soybase, *qPH14_1* in Y159 population might be a new QTL regulating plant height. Four QTL for branch number were identified in Y32 and Y133 populations. Six QTL for node numbers of main stem were detected in Y133 and Y159 populations, the QTL *qNode6_1* in Y133 population was near the reported loci with about 43.96 kb, and the other five QTL were not reported. Six QTL for pods per plant were detected in three populations, *qPod1_1* of Y32, *qPod19_1* of Y133 and *qPod20_1* of Y159 were three new QTL loci. These QTL mapping can provide a theoretical basis for further genetic analysis of these important traits, enrich QTL loci for important traits, and promote marker-assisted breeding.

## Conclusions

A total of 10 QTL for flowering time were identified in three biparental populations. Some QTL were corresponding to the *E1* or *E2* genes or the other reported QTL. In fact, the *E1* and *E2* genes may be detected when the parents carry different *E1* and *E2* genotypes indicating that the major *E* loci have a significant influence on flowering time. However, the effect of *E1* and *E2* genes on flowering time are different under various *E* genes background. In Y159 population, QTL of *qFT4_1* and *qFT4_2* on chromosome 4 were new QTL for flowering time. In order to clone some minor QTL loci, the population was divided into sub-populations with the same genotype of *E* genes, and some additional QTL with high PVE were identified. Meanwhile, compared with the QTL reported in Soybase, 1 QTL for plant height (PH), 3 QTL for branch number (BR), 5 QTL for node numbers of main stem, and 3 QTL for pods per plant might be new QTL.

## Supplemental Information

10.7717/peerj.12416/supp-1 Supplemental Information 1 Statistics of sequencing data of three F_2_ populations.

10.7717/peerj.12416/supp-2 Supplemental Information 2 Number of SLAF marker in three F_2_ populations.

10.7717/peerj.12416/supp-3 Supplemental Information 3 Identification of additional QTL by removing the effect of *E1* gene in Y32 population.

10.7717/peerj.12416/supp-4 Supplemental Information 4 Identification of additional QTL by removing the effect of *E1* gene in Y133 population.

10.7717/peerj.12416/supp-5 Supplemental Information 5 Identification of additional QTL by removing the effect of *E2* gene in Y159 population.

10.7717/peerj.12416/supp-6 Supplemental Information 6 Distribution of SLAF labels and polymorphism SLAF labels on twenty chromosomes in three F_2_ populations.

10.7717/peerj.12416/supp-7 Supplemental Information 7 The collinearity analysis of twenty linkage maps with the soybean reference genome in three F_2_ populations.

10.7717/peerj.12416/supp-8 Supplemental Information 8 The genotype of *E1* and *E2* of six parents (M: 2,000 bp marker; L1: Xudou 9; L2: Daheiqi; L3: Kenfeng 16; L4: Pixian ruantiaozhi; L5: Liaodou 15; L6: Jilin 35).

10.7717/peerj.12416/supp-9 Supplemental Information 9 Phenotypic data of Y32 population.

10.7717/peerj.12416/supp-10 Supplemental Information 10 Phenotypic data of Y133 population.

10.7717/peerj.12416/supp-11 Supplemental Information 11 Poly SLAF markers of Y32 population.

10.7717/peerj.12416/supp-12 Supplemental Information 12 Phenotypic data of Y159 population.

10.7717/peerj.12416/supp-13 Supplemental Information 13 Poly SLAF markers of Y133 population.

10.7717/peerj.12416/supp-14 Supplemental Information 14 Poly SLAF markers of Y159 population.

10.7717/peerj.12416/supp-15 Supplemental Information 15 Supplemental Tables.

## Additional Information and Declarations

### Data Availability

The following information was supplied regarding data availability: Phenotypic data are available in the Supplemental Files. The data is available at National Genomics Data Center: PRJCA002955.

## References

[ref-1] Assefa T, Otyama PI, Brown AV, Kalberer SR, Kulkarni RS, Cannon SB (2019). Genome-wide associations and epistatic interactions for internode number, plant height, seed weight and seed yield in soybean. BMC Genomics.

[ref-2] Board JE, Tan Q (1995). Assimilatory capacity effects on soybean yield components and pod number. Crop Science.

[ref-3] Ca YG, Li SG, He XH, Chang FG, Kong JJ, Gai JY, Zhao TJ (2017). Mapping QTLs for plant height and flowering time in a Chinese summer planting soybean RIL population. Euphytica.

[ref-4] Chen QS, Zhang ZC, Liu CY, Xin DW, Qiu HM, Shan DP, Shan CY, Hu GH (2007). QTL analysis of major agronomic traits in soybean. Agricultural Sciences in China.

[ref-5] Fehr WR, Caviness CE, Burmood DT, Penningt J (1971). Stage of development descriptions for soybeans, *Glycine-max* (L) Merrill. Crop Science.

[ref-49] Funatsuki H, Kawaguchi K, Matsuba S, Sato Y, Ishimoto M (2005). Mapping of QTL associated with chilling tolerance during reproductive growth in soybean. Theoretical and Applied Genetics.

[ref-6] He QY, Yang HY, Xiang SH, Wang WB, Xing GN, Zhao TJ, Gai JY (2014). QTL mapping for the number of branches and pods using wild chromosome segment substitution lines in soybean (*Glycine max L*.). Plant Genetic Resourcees Ccharacteerization and Utilizatioon.

[ref-7] Jiang BJ, Nan HY, Gao YF, Tang LL, Yue YL, Lu SJ, Ma LM, Cao D, Sun S, Wang JL, Wu CX, Yuan XH, Hou WS, Kong FJ, Han TF, Liu BH (2014). Allelic combinations of Soybean maturity loci *E1*, *E2*, *E3* and *E4* result in diversity of maturity and adaptation to different latitudes. PLOS ONE.

[ref-50] Kabelka EA, Diers BW, Fehr WR, LeRoy AR, Baianu IC, You T, Neece DJ, Nelson RL (2004). Putative alleles for increased yield from soybean plant introductions. Crop Science.

[ref-8] Kong FJ, Liu BH, Xia ZJ, Sato S, Kim BM, Watanabe S, Yamada T, Tabata S, Kanazawa A, Harada K, Abe J (2010). Two coordinately regulated homologs of *FLOWERING LOCUS T* are involved in the control of photoperiodic flowering in soybean. Plant Physiology.

[ref-9] Kong FJ, Nan HY, Cao D, Li Y, Wu FF, Wang JL, Lu SJ, Yuan XH, Cober ER, Abe J, Liu BH (2014). A new gene *E9* conditions early flowering and maturity in soybean. Crop Science.

[ref-43] Kuroda Y, Kaga A, Norihiko T, Yano H, Kato S, Vaughan D (2013). QTL affecting fitness of hybrids between wild and cultivated soybeans in experimental fields. Ecology and Evolution.

[ref-10] Langewisch T, Lenis J, Jiang GL, Wang DC, Pantalone V, Bilyeu K (2017). The development and use of a molecular model for soybean maturity groups. BMC Plant Biology.

[ref-11] Li YQ, Dong YS, Wu HY, Hu B, Zhai H, Yang JY, Xia ZJ (2019). Positional cloning of the flowering time QTL *qFT12-1* reveals the link between the clock related *PRR* homolog with photoperiodic response in soybeans. Frontiers in Plant Science.

[ref-12] Li B, Tian L, Zhang JY, Huang L, Han FX, Yan SR, Wang LZ, Zheng HK, Sun JM (2014). Construction of a high-density genetic map based on large-scale markers developed by specific length amplified fragment sequencing (SLAF-seq) and its application to QTL analysis for isoflavone content in *Glycine max*. BMC Genomics.

[ref-13] Li JC, Wang XB, Song WW, Huang XY, Zhou J, Zeng HY, Sun S, Jia HC, Li WB, Zhou XN, Li SZ, Chen PY, Wu CX, Guo Y, Han TF, Qiu LJ (2017). Genetic variation of maturity groups and four *E* genes in the Chinese soybean mini core collection. PLOS ONE.

[ref-14] Li H, Ye G, Wang J (2007). A modified algorithm for the improvement of composite interval mapping. Genetics.

[ref-15] Li WX, Zheng DH, Van K, Lee SH (2008). QTL mapping for major agronomic traits across two years in soybean (*Glycine max L. Merr*). Journal of Crop Science and Biotechnology.

[ref-16] Liu BH, Kanazawa A, Matsumura H, Takahashi R, Harada K, Abe J (2008). Genetic redundancy in soybean photoresponses associated with duplication of the phytochrome A gene. Genetics.

[ref-45] Liu W, Kim M, Van K, Lee Y, Li H, Liu X, Lee S (2011). QTL identification of yield-related traits and their association with flowering and maturity in soybean. Journal of Crop Science and Biotechnology.

[ref-17] Liu DY, Ma CX, Hong WG, Huang L, Liu M, Liu H, Zeng HP, Deng DJ, Xin HG, Song J, Xu CH, Sun XW, Hou XL, Wang XW, Zheng HK (2014). Construction and analysis of high-density linkage map using high-throughout sequencing data. PLOS ONE.

[ref-18] Liu YL, Reif JC, Mette MF, Liu ZX, Liu B, Zhang SS, Yan L, Chang RZ, Qiu LJ (2013). Identification of quantitative trait loci underlying plant height and seed weight in soybean. The Plant Genome.

[ref-19] Lu SJ, Ying Li, Wang JL, Nan HY, Cao D, Li XM, Shi DN, Fang C, Shi XY, Yuan XH, Jun A, Liu BH, Kong FJ (2016). Identification of additional QTLs for flowering time by removing the effect of the maturity gene *E1* in soybean. Journal of Integrative Agriculture.

[ref-20] Lu SJ, Zhao XH, Hu Y, Liu S, Nan HY, Li X, Fang C, Cao D, Shi X, Kong LP, Su T, Zhang F, Li SC, Wang Z, Yuan XH, Cober ER, Weller JL, Liu BH, Hou X, Tian ZX, Kong FJ (2017). Natural variation at the soybean *j* locus improves adaptation to the tropics and enhances yield. Nature Genetics.

[ref-21] Murray MG, Thompson WF (1980). Rapid isolation of high molecular-weight plant DNA. Nucleic Acids Research.

[ref-48] Orf JH, Chase K, Jarvik T, Mansur LM, Cregan PB, Adler FR, Lark KG (1999). Genetics of soybean agronomic traits: I. Comparison of three related recombinant inbred populations. Crop Science.

[ref-22] Panthee DR, Pantalone VR, Saxton AM, West DR, Sams CE (2007). Quantitative trait loci for agronomic traits in soybean. Plant Breeding.

[ref-23] Qi ZM, Huang L, Zhu RS, Xin DW, Liu CY, Han X, Jiang HW, Hong WG, Hu GH, Zheng HK, Chen QS (2014). A high-density genetic map for soybean based on specific length amplified fragment sequencing. PLOS ONE.

[ref-51] Reinprecht Y, Poysa V, Yu K, Rajcan I, Ablett G, Pauls K (2006). Seed and agronomic QTL in low linolenic acid, lipoxygenase-free soybean (Glycine max (L.) Merrill) germplasm. Genome.

[ref-24] Sayama TH, Wang TY, Yamazaki H, Yamaguchi N, Komatsu K, Takahashi M, Suzuki C, Miyoshi T, Tanaka Y, Xiao ZJ, Tsubokura Y, Watanabe S, Harada K, Funatsuki H, Ishimoto M (2010). Mapping and comparison of quantitative trait loci for soybean branching phenotype in two locations. Breeding Science.

[ref-46] Specht JE, Chase K, Macrander M, Graef GL, Chung J, Markwell JP, Germann M, Orf JH, Lark KG (2001). Soybean response to water: a QTL analysis of drought tolerance. Crop Science.

[ref-25] Stam P (1993). Construction of integrated genetic linkage maps by means of a new computer package: JOINMAP. The Plant Journal.

[ref-26] Sun XW, Liu DY, Zhang XF, Li WB, Liu H, Hong WG, Jiang CB, Guan N, Ma CX, Zeng HP, Xu CH, Song J, Huang L, Wang CM, Shi JJ, Wang R, Zheng XH, Lu CY, Wang XW, Zheng HK (2013). SLAF-seq: An efficient method of large-scale De novo SNP discovery and genotyping using high-throughput sequencing. PLOS ONE.

[ref-42] Tasma IM, Lorenzen LL, Green DE, Shoemaker RC (2001). Mapping genetic loci for flowering time, maturity, and photoperiod insensitivity in soybean. Molecular Breeding.

[ref-47] Vieira A, Oliveira A, Soares T, Schuster I, Piovesan N, Martinez C, Barros E, Moreira M (2006). Use of the QTL approach to the study of soybean trait relationships in two populations of recombinant inbred lines at the F7 and F8 generations. Brazilian Journal of Physics.

[ref-27] Wang YY, Li YQ, Wu HY, Hu B, Zheng JJ, Zhai H, Lu SX, Liu XL, Chen X, Qiu HM, Yang JY, Zong CM, Han DZ, Wen ZX, Wang DC, Xia ZJ (2018). Genotyping of soybean cultivars with medium-density array reveals the population structure and QTNs underlying maturity and seed traits. Frontiers in Plant Science.

[ref-28] Wang FF, Nan HY, Chen LY, Fang C, Zhang HY, Su T, Li SC, Cheng Q, Dong LD, Liu BH, Kong FJ, Lu SJ (2019). A new locus, *E11*, controls early flowering time and maturity in soybean. Molecular Breeding.

[ref-29] Watanabe S, Hideshima R, Xia ZJ, Tsubokura Y, Sato S, Nakamoto Y, Yamanaka N, Takahashi R, Ishimoto M, Anai T, Tabata S, Harada K (2009). Map-based cloning of the gene associated with the soybean maturity locus *E3*. Genetics.

[ref-30] Watanabe S, Xia ZJ, Hideshima R, Tsubokura Y, Sato S, Yamanaka N, Takahashi R, Anai T, Tabata S, Kitamura K, Harada K (2011). A map-based cloning strategy employing a residual heterozygous line reveals that the *GIGANTEA* gene is involved in soybean maturity and flowering. Genetics.

[ref-52] Watanabe S, Harada K, Abe J (2012). Genetic and molecular bases of photoperiod responses of flowering in soybean. Breeding Sciences.

[ref-31] Wilcox JR (2004). World distribution and trade of soybean. Soybeans.

[ref-32] Xia ZJ, Tsubokura Y, Hoshi M, Hanawa M, Yano C, Okamura K, Ahmed TA, Anai T, Watanabe S, Hayashi M, Kawai T, Hossain KG, Masaki H, Asai K, Yamanaka N, Kubo N, Kadowaki KI, Nagamura Y, Yano M, Sasaki T (2007). An integrated high-density linkage map of soybean with RFLP, SSR, STS, and AFLP markers using a single F2 population. DNA Research.

[ref-33] Xia ZJ, Watanabe S, Yamada T, Tsubokura S, Nakashima H, Zhai H, Anai T, Sato S, Yamazaki T, Lü SX, Wu HY, Tabata S, Harada K (2012). Positional cloning and characterization reveal the molecular basis for soybean maturity locus *E1*, which regulates photoperiodic flowering. Proceedings of the National Academy of Sciences of the United States of America.

[ref-34] Xu ML, Xu Z, Liu BH, Kong FJ, Tsubokura Y, Watanabe S, Xia ZJ, Harada K, Kanazawa A, Yamada T, Abe J (2013). Genetic variation in four maturity genes affects photoperiod insensitivity and PHYA-regulated post flowering responses of soybean. BMC Plant Biology.

[ref-35] Yang G, Zhai H, Wu HY, Zhang XZ, Lu SX, Wang YY, Li YQ, Hu B, Wang L, Wen ZX, Wang DC, Wang SD, Harada K, Xia ZJ, Xie FT (2017). QTL effects and epistatic interaction for flowering time and branch number in soybean mapping population of Japanese×Chinese cultivars. Journal of Integrative Agriculture.

[ref-44] Yao D, Liu Z, Zhang J, Liu S, Qu J, Guan S, Pan L, Wang D, Liu J, Wang P (2015). Analysis of quantitative trait loci for main plant traits in soybean. Genetics and Molecular Research.

[ref-36] Yin ZG, Qi HD, Mao XR, Wang JX, Hu ZB, Wu XX, Liu CY, Xin DW, Zuo X, Chen QS, Qi ZM (2018). QTL mapping of soybean node numbers on the main stem and meta-analysis for mining candidate genes. Biotechnology & Biotechnological Equipment.

[ref-37] Zhai H, Lü SX, Liang S, Wu HY, Zhang XZ, Liu BH, Kong FJ, Yuan XH, Li J, Xia ZJ (2014a). *GmFT4*, a homolog of *FLOWERING LOCUS T*, is positively regulated by *E1* and functions as a flowering repressor in soybean. PLOS ONE.

[ref-38] Zhai H, Lü SX, Wang YQ, Chen X, Ren HX, Yang JY, Cheng W, Zong CM, Gu HP, Qiu HM, Wu HY, Zhang XZ, Cui TT, Xia ZJ (2014b). Allelic variations at four major maturity *E* genes and transcriptional abundance of the *E1* gene are associated with flowering time and maturity of soybean cultivars. PLOS ONE.

[ref-39] Zhang D, Cheng H, Wang H, Zhang HY, Liu CY, Yu DY (2010). Identification of genomic regions determining flower and pod numbers development in soybean (*Glycine max L*.). Journal of Genetics and Genomics.

[ref-40] Zhang YW, Li W, Lin YH, Zhang LF, Wang CJ, Xu R (2018). Construction of a high-density genetic map and mapping of QTLs for soybean (*Glycine max*) agronomic and seed quality traits by specific length amplified fragment sequencing. BMC Genomics.

[ref-41] Zhang WK, Wang YJ, Luo GZ, Zhang JS, He CY, Wu XL, Gai JY, Chen SY (2004). QTL mapping of ten agronomic traits on the soybean (*Glycine max L. Merr*) genetic map and their association with EST markers. Theoretical and Applied Genetics.

