# Supplementary material for: Identification of quantitative trait loci underlying five major agronomic traits of soybean in three biparental populations by specific length amplified fragment sequencing (SLAF-seq)
Source: PeerJ. 2021 Dec 14;9:e12416. doi: 10.7717/peerj.12416 (PMC8679901; doi:10.7717/peerj.12416)
Supplement: Supplemental Information 1 [file peerj-09-12416-s001.pdf]

Table S1 Statistics of sequencing data of three $F_2$ populations.

| Population | Total Reads | Total Bases | Q30 Percentage (%) | GC Percentage (%) |
|---|---|---|---|---|
| Y32 | 162880924 | 32543922562 | 84.65 | 41.71 |
| Y133 | 148125690 | 29606583860 | 90.15 | 42.39 |
| Y159 | 167249759 | 33425150914 | 84.65 | 41.58 |
