# Supplementary material for: Identification of quantitative trait loci underlying five major agronomic traits of soybean in three biparental populations by specific length amplified fragment sequencing (SLAF-seq)
Source: PeerJ. 2021 Dec 14;9:e12416. doi: 10.7717/peerj.12416 (PMC8679901; doi:10.7717/peerj.12416)
Supplement: Supplemental Information 2 [file peerj-09-12416-s002.pdf]

Table S2 Number of SLAF marker in three F_2 populations.

Population	Total SLAF	Polymorphic SLAF	Percentage	SLAF Number
Y32	160979	27430	17.04%	6872
Y133	141341	17915	12.68%	7136
Y159	156180	2178	13.95%	7924
