# Supplementary material for: Identification of quantitative trait loci underlying five major agronomic traits of soybean in three biparental populations by specific length amplified fragment sequencing (SLAF-seq)
Source: PeerJ. 2021 Dec 14;9:e12416. doi: 10.7717/peerj.12416 (PMC8679901; doi:10.7717/peerj.12416)
Supplement: Supplemental Information 3 [file peerj-09-12416-s003.pdf]

Table S3 Identification of additional QTL by removing the effect of *E1* gene in Y32 population.

Populatior	QTL	Chr	LeftMarker	Physical position (bp)	RightMarker	Physical position (bp)	LOD	PVE(%)	Add	Dom	Distance to known QTL or gene (kb)	QTL in SoyBase or known gene
**Y32-*E1***	*qFT10_2*	10	Marker776629	45046804	Marker863420	45440450	3.09	43.92	1.96	-9.65	Included	*E2* (Watanabe et al., 2011)
	qFT20_2	20	Marker1228966	1956141	Marker1135381	1795026	3.05	44.62	1.08	7.42		
	qPH9_1	9	Marker669769	5584243	Marker623942	6496253	6.18	67.61	-6.32	-72.55	55.48kb	Plant height 17-4 (Yao et al., 2015A)
	qBR10_1	10	Marker811909	43784166	Marker748599	44987192	6.97	49.74	-0.08	-4.05	273.27kb	Branching 2-1 (Li et al., 2008)
	qBR15_1	15	Marker2274799	50732054	Marker2310522	1742838	5.59	33.30	-1.16	-0.09	Included	Branching 1-5 (Chen et al., 2007)
	qBR20_1	20	Marker1139561	38365623	Marker1161006	37006103	3.93	19.98	-0.91	0.05		
	qNode15_1	15	Marker2229610	11939150	Marker2254444	12382773	19.40	98.17	77.59	-76.71		
	qPod1_2	1	Marker2898538	4648217	Marker2846473	4782894	5.68	50.33	65.58	10.24		
	qPod12_1	12	Marker1358768	5868300	Marker1454425	4981513	2.97	19.74	5.83	68.58		
	qPod19_1	19	Marker2376336	38154884	Marker2480383	37869958	3.33	13.09	11.37	43.08		
**Y32-*e1***	*qFT4_1*	4	Marker3295408	4553895	Marker3288456	3168820	3.84	66.10	-7.19	6.15		
	qFT5_1	5	Marker1493500	39601969	Marker1488945	41318423	3.33	55.25	-0.40	-9.34		
	qFT8_1	8	Marker444770	46542606	Marker403828	45027949	2.66	67.92	-6.32	8.15		
	qPH2_1	2	Marker1983460	12924730	Marker1830524	9900064	4.43	31.03	13.11	-25.74	417.73kb	Plant height 23-1 (Reinprecht et al., 2006)
	qPH2_2	2	Marker1874132	39089204	Marker1930951	39464576	2.95	23.15	11.85	-17.18	868.18kb	Plant height 33-3 (Kim et al., 2012)
	qPH4_1	4	Marker3295408	4553895	Marker3288456	3168820	3.05	27.30	-6.88	26.19	132kb	Plant height 33-4 (Kim et al., 2012)
	qPH8_1	8	Marker503095	44453489	Marker468911	44291501	2.89	23.62	-10.77	19.26		
	qPH13_1	13	Marker969634	23067750	Marker1072088	21754282	4.29	33.91	13.94	-25.73	Inside	Plant height 37-8 (Yao et al., 2015)
	qPH14_1	14	Marker1762040	7057513	Marker1709446	4700609	2.86	29.48	14.83	-26.94	2595.82kb	Plant height 34-6 (Kim et al., 2012)
	qPH15_3	15	Marker2188521	51667997	Marker2193874	50669471	3.77	27.57	12.16	-21.59	352.24kb	Plant height 13-3 (Specht et al., 2001)
	qPH15_4	15	Marker2256742	10683143	Marker2248389	6350450	3.44	45.87	-3.98	-60.26		
	qBR10_2	10	Marker753141	2569603	Marker871220	2811723	2.82	13.57	1.02	-0.50		
	qBR10_3	10	Marker856739	2496414	Marker881068	1530799	2.95	14.14	1.08	-0.38		
	qBR11_1	11	Marker2102110	5764052	Marker2056824	4504984	2.65	12.81	0.91	-1.07	Inside	Branching 1-1 (Chen et al., 2007)
	qBR13_1	13	Marker1045635	33388825	Marker1094632	32571480	2.95	23.45	0.13	-2.67		
	qBR14_1	14	Marker1792639	38716712	Marker1643198	44087851	4.68	35.73	1.71	-1.38		
	qNode3_1	3	Marker51677	34432502	Marker85007	35368824	11.44	26.12	0.13	-4.46		
	qNode3_2	3	Marker34365	41212483	Marker37993	44054643	8.82	14.82	-0.17	2.97		
	qNode9_1	9	Marker725322	1573416	Marker656516	789646	4.28	4.67	0.02	2.94		
	qNode15_2	15	Marker2256742	10683143	Marker2248389	6350450	17.39	83.82	0.04	-12.54		
	qNode20_1	20	Marker1112314	2032772	Marker1291840	23479729	7.42	11.07	-1.53	0.04		
	qPod2_1	2	Marker1894321	42679545	Marker1907855	43898375	3.49	49.30	68.55	-42.72		
