# Supplementary material for: Identification of quantitative trait loci underlying five major agronomic traits of soybean in three biparental populations by specific length amplified fragment sequencing (SLAF-seq)
Source: PeerJ. 2021 Dec 14;9:e12416. doi: 10.7717/peerj.12416 (PMC8679901; doi:10.7717/peerj.12416)
Supplement: Supplemental Information 4 [file peerj-09-12416-s004.pdf]

Table S4 Identification of additional QTL by removing the effect of *E1* gene in Y133 population.

Population	QTL	Chr	LeftMarker	Physical position (bp)	RightMarker	Physical position (bp)	LOD	PVE(%)	Add	Dom	Distance to known QTL or gene (kb)	QTL in SoyBase or known gene
Y133-*E1*	*qFT1_1*	1	Marker860296	49059102	Marker889667	48966442	4.92	14.37	-2.53	-0.56		
	qFT6_2	6	Marker1449694	46856851	Marker1596115	45088674	7.36	29.52	-4.08	0.58	762.59kb	First flower 1-2 (Keim et al., 1990)
	qFT7_2	7	Marker1838814	42480669	Marker1823729	43139642	3.70	4.88	1.76	1.38		
	qFT18_2	18	Marker2566070	2491200	Marker2566544	2374218	3.30	8.84	0.12	3.11	624.28kb	First flower 21-4 (Reinprecht et al., 2006)
	qFT20_2	20	Marker1380631	43548635	Marker1408825	47015159	10.50	58.84	-0.17	-9.06		
	qPH19_2	19	Marker1629638	44862177	Marker1666593	45184768	3.20	41.35	5.45	29.49	0.23kb	Plant height 10-4 (Orf et al., 1999)
	qPH19_3	19	Marker1620683	40760282	Marker1769282	40958504	2.98	42.27	14.61	1.26	578.02kb	Plant height 8-3 (Orf et al., 1999)
	qBR2_1	2	Marker949955	11687058	Marker1059871	11367075	3.06	14.01	0.61	2.11		
	qBR5_1	5	Marker3267895	34369728	Marker3340114	31545456	3.04	19.06	-0.83	-2.34	Inside	Branching 4-1 (Yao et al., 2015)
	qBR6_1	6	Marker1570324	17656443	Marker1578129	16421211	4.72	45.32	-2.29	1.18	1899.28kb	Branching 3-1 (Sayama et al., 2010)
	qBR7_1	7	Marker1835020	37550684	Marker1860076	3572434	3.26	27.27	-1.80	0.30		
	qBR8_1	8	Marker2713366	40908851	Marker2800357	38375557	2.61	12.51	-0.97	0.38		
	qNode6_2	6	Marker1490011	9213156	Marker1524454	8060722	2.85	25.30	0.53	-3.05		
	qNode6_3	6	Marker1523371	5747476	Marker1535031	3027287	3.08	31.93	0.20	-3.25		
	qNode8_1	8	Marker2713366	40908851	Marker2800357	38375557	2.90	41.84	-1.81	1.21		
	qNode9_1	9	Marker3118242	2275187	Marker3120808	1116389	2.51	22.93	-1.32	1.08		
	qNode20_1	20	Marker1258177	37795328	Marker1320117	40695692	2.67	24.37	0.34	-2.71		
	qPod11_2	11	Marker1183720	6021949	Marker1210060	4627307	2.76	23.99	7.07	64.82	340kb	Pod number 3-1 (Sun et al., 2006)
	qPod15_2	15	Marker476641	5187960	Marker330620	4141915	2.56	22.82	22.47	-45.67		
	qPod19_2	19	Marker1635921	41294702	Marker1620683	40760479	3.04	43.48	43.31	-24.94	321.49kb	Pod number 9-3 (Kuroda et al., 2013)
Y133-*e1*	*qFT6_3*	6	Marker1550448	43529248	Marker1596115	45088674	7.62	32.58	-2.49	-11.72	461.41kb	First flower 12-1 (Zhang et al., 2004)
	qFT6_4	6	Marker1547068	11086775	Marker1452469	10820805	3.08	4.52	-2.30	-3.35		
	qFT6_5	6	Marker1523034	5949576	Marker1536475	3149103	3.86	11.93	3.57	-0.66		
	qFT16_1	16	Marker2199160	175187	Marker2153289	119447	7.36	34.73	-0.60	12.36		
	qPH6_1	6	Marker1434937	15504018	Marker1558850	13723869	2.94	47.72	-20.36	-24.52		
	qPH8_1	8	Marker2711396	17240120	Marker2770106	15919776	2.55	52.93	-1.06	45.74		
	qPH15_1	15	Marker394633	14911800	Marker480088	17330244	3.40	56.64	11.74	-36.26	1257.82kb	Plant height 29-4 (Liu et al., 2011)
	qBR14_1	14	Marker2886171	345481	Marker2833216	452968	2.57	43.23	-1.73	-0.53		
	qBR15_1	15	Marker481532	1825138	Marker437611	1984973	2.84	46.43	0.65	2.66		
	qBR17_1	17	Marker52634	11719926	Marker98971	12110861	3.56	54.16	-1.35	-1.50	Inside	Branching 3-4 (Sayama et al., 2010)
	qBR17_2	17	Marker134981	37768211	Marker100052	38188111	3.18	50.60	-1.37	-2.53		
	qNode4_1	4	Marker649171	5294909	Marker673149	1650768	3.04	7.65	-1.26	-1.69		
	qNode6_4	6	Marker1587916	34230280	Marker1561476	44179958	5.37	30.58	0.34	-5.33	189.3kb	Node number 1-4 (Gai et al., 2007)
	qNode9_2	9	Marker3125692	8507082	Marker3083552	8701969	2.55	5.88	-0.41	1.73		
	qNode10_1	10	Marker2050793	51561569	Marker2002012	49350327	2.69	6.12	0.36	2.45		
	qNode15_1	15	Marker318422	9999755	Marker394993	14823774	5.35	31.79	0.04	-5.55		
	qNode16_2	16	Marker2199160	175187	Marker2153289	119447	3.40	8.34	-1.28	0.00		
	qNode17_1	17	Marker130433	7779356	Marker135800	5852893	4.23	8.89	-0.15	2.29	Inside	Node number 7-1 (Li et al., 2009)
	qNode19_2	19	Marker1695229	42565531	Marker1722002	44921078	3.25	14.37	0.22	2.74		
	qPod11_2	11	Marker1191778	4626607	Marker1158449	4567661	2.82	52.77	20.64	56.72		
	qPod17_1	17	Marker71932	4835831	Marker57299	3850128	2.54	42.84	-26.06	-6.53		
