# Supplementary material for: Identification of quantitative trait loci underlying five major agronomic traits of soybean in three biparental populations by specific length amplified fragment sequencing (SLAF-seq)
Source: PeerJ. 2021 Dec 14;9:e12416. doi: 10.7717/peerj.12416 (PMC8679901; doi:10.7717/peerj.12416)
Supplement: Supplemental Information 5 [file peerj-09-12416-s005.pdf]

Table S5 Identification of additional QTL by removing the effect of *E2* gene in Y159 population.

Population	QTL	Chr	LeftMarker	Physical position (bp)	RightMarker	Physical position (bp)	LOD	PVE(%)	Add	Dom	Distance to known QTL or gene (kb)	QTL in SoyBase or known gene
Y159-*E2*	*qFT16_1*	16	Marker133751	1744685	Marker4884	1469283	2.55	19.34	0.85	5.78		
	qFT18_1	18	Marker2873468	8587965	Marker2951053	7774568	4.00	60.20	4.25	-1.77	742.96kb	First flower 9-2 (Reinprecht et al., 2006)
	qPH2_1	2	Marker2073773	43210350	Marker1997222	41732879	3.09	74.12	6.56	13.54	700.31kb	Plant height 26-9 (Sun et al., 2006)
	qPH4_1	4	Marker2251035	41180835	Marker2328095	13336223	2.62	45.61	10.64	16.83		
	qBR2_1	2	Marker2084568	285567	Marker2046426	1200000	3.85	58.91	-2.34	-3.49		
	qBR4_1	4	Marker2211959	14076044	Marker2180729	12902708	3.60	57.78	-1.88	-4.37		
	qBR10_1	10	Marker2491207	46582574	Marker2562044	48212365	3.73	64.74	0.46	4.42		
	qBR12_1	12	Marker259797	39092894	Marker190876	2635815	2.63	58.77	-0.53	4.81	Included	Branching 5-3 (Shim et al., 2018)
	qNode2_1	2	Marker2004129	38592420	Marker2057410	38154226	4.29	2.12	-0.54	-0.06	66.8kb	Node number 4-1 (Liu et al., 2011)
	qNode4_1	4	Marker2263186	3853836	Marker2194298	3567846	9.25	9.34	0.02	-2.46		
	qNode9_1	9	Marker1445830	49555447	Marker1384932	48059714	8.28	7.48	0.01	1.93		
	qNode10_1	10	Marker2581131	45301061	Marker2607150	46746333	15.76	43.82	4.91	-4.43		
	qPod12_1	12	Marker160225	34446165	Marker277796	37051858	2.74	55.29	-18.58	78.28		
