# Supplementary material for: Identification of quantitative trait loci underlying five major agronomic traits of soybean in three biparental populations by specific length amplified fragment sequencing (SLAF-seq)
Source: PeerJ. 2021 Dec 14;9:e12416. doi: 10.7717/peerj.12416 (PMC8679901; doi:10.7717/peerj.12416)
Supplement: Supplemental Information 15 [file peerj-09-12416-s015.docx]

| Table S1 Statistics of sequencing data of three F_2_ populations. | | | |  |
| --- | --- | --- | --- | --- |
| Population | Total Reads | Total Bases | Q30 Percentage (%) | GC Percentage (%) |
| Y32 | 162880924 | 32543922562 | 84.65 | 41.71 |
| Y133 | 148125690 | 29606583860 | 90.15 | 42.39 |
| Y159 | 167249759 | 33425150914 | 84.65 | 41.58 |

| Table S2 Number of SLAF marker in three F_2_ populations. | | | |  |
| --- | --- | --- | --- | --- |
| Population | Total SLAF | Polymorphic SLAF | Percentage | SLAF Number |
| Y32 | 160979 | 27430 | 17.04% | 6872 |
| Y133 | 141341 | 17915 | 12.68% | 7136 |
| Y159 | 156180 | 2178 | 13.95% | 7924 |

| Table S3 Identification of additional QTL by removing the effect of *E1* gene in Y32 population. | | | | | | |  |  |  |  |  |  |
| --- | --- | --- | --- | --- | --- | --- | --- | --- | --- | --- | --- | --- |
| Population | QTL | Chr | LeftMarker | Physical position (bp) | RightMarker | Physical position (bp) | LOD | PVE(%) | Add | Dom | Distance to known QTL or gene (kb) | QTL in SoyBase or known gene |
| **Y32-*E1*** | *qFT10_2* | 10 | Marker776629 | 45046804 | Marker863420 | 45440450 | 3.09 | 43.92 | 1.96 | -9.65 | Included | *E2* (Watanabe et al., 2011) |
|  | *qFT20_2* | 20 | Marker1228966 | 1956141 | Marker1135381 | 1795026 | 3.05 | 44.62 | 1.08 | 7.42 |  |  |
|  | *qPH9_1* | 9 | Marker669769 | 5584243 | Marker623942 | 6496253 | 6.18 | 67.61 | -6.32 | -72.55 | 55.48kb | Plant height 17-4 (Yao et al., 2015A) |
|  | *qBR10_1* | 10 | Marker811909 | 43784166 | Marker748599 | 44987192 | 6.97 | 49.74 | -0.08 | -4.05 | 273.27kb | Branching 2-1 (Li et al., 2008) |
|  | *qBR15_1* | 15 | Marker2274799 | 50732054 | Marker2310522 | 1742838 | 5.59 | 33.30 | -1.16 | -0.09 | Included | Branching 1-5 (Chen et al., 2007) |
|  | *qBR20_1* | 20 | Marker1139561 | 38365623 | Marker1161006 | 37006103 | 3.93 | 19.98 | -0.91 | 0.05 |  |  |
|  | *qNode15_1* | 15 | Marker2229610 | 11939150 | Marker2254444 | 12382773 | 19.40 | 98.17 | 77.59 | -76.71 |  |  |
|  | *qPod1_2* | 1 | Marker2898538 | 4648217 | Marker2846473 | 4782894 | 5.68 | 50.33 | 65.58 | 10.24 |  |  |
|  | *qPod12_1* | 12 | Marker1358768 | 5868300 | Marker1454425 | 4981513 | 2.97 | 19.74 | 5.83 | 68.58 |  |  |
|  | *qPod19_1* | 19 | Marker2376336 | 38154884 | Marker2480383 | 37869958 | 3.33 | 13.09 | 11.37 | 43.08 |  |  |
| **Y32-*e1*** | *qFT4_1* | 4 | Marker3295408 | 4553895 | Marker3288456 | 3168820 | 3.84 | 66.10 | -7.19 | 6.15 |  |  |
|  | *qFT5_1* | 5 | Marker1493500 | 39601969 | Marker1488945 | 41318423 | 3.33 | 55.25 | -0.40 | -9.34 |  |  |
|  | *qFT8_1* | 8 | Marker444770 | 46542606 | Marker403828 | 45027949 | 2.66 | 67.92 | -6.32 | 8.15 |  |  |
|  | *qPH2_1* | 2 | Marker1983460 | 12924730 | Marker1830524 | 9900064 | 4.43 | 31.03 | 13.11 | -25.74 | 417.73kb | Plant height 23-1 (Reinprecht et al., 2006) |
|  | *qPH2_2* | 2 | Marker1874132 | 39089204 | Marker1930951 | 39464576 | 2.95 | 23.15 | 11.85 | -17.18 | 868.18kb | Plant height 33-3 (Kim et al., 2012) |
|  | *qPH4_1* | 4 | Marker3295408 | 4553895 | Marker3288456 | 3168820 | 3.05 | 27.30 | -6.88 | 26.19 | 132kb | Plant height 33-4 (Kim et al., 2012) |
|  | *qPH8_1* | 8 | Marker503095 | 44453489 | Marker468911 | 44291501 | 2.89 | 23.62 | -10.77 | 19.26 |  |  |
|  | *qPH13_1* | 13 | Marker969634 | 23067750 | Marker1072088 | 21754282 | 4.29 | 33.91 | 13.94 | -25.73 | Inside | Plant height 37-8 (Yao et al., 2015) |
|  | *qPH14_1* | 14 | Marker1762040 | 7057513 | Marker1709446 | 4700609 | 2.86 | 29.48 | 14.83 | -26.94 | 2595.82kb | Plant height 34-6 (Kim et al., 2012) |
|  | *qPH15_3* | 15 | Marker2188521 | 51667997 | Marker2193874 | 50669471 | 3.77 | 27.57 | 12.16 | -21.59 | 352.24kb | Plant height 13-3 (Specht et al., 2001) |
|  | *qPH15_4* | 15 | Marker2256742 | 10683143 | Marker2248389 | 6350450 | 3.44 | 45.87 | -3.98 | -60.26 |  |  |
|  | *qBR10_2* | 10 | Marker753141 | 2569603 | Marker871220 | 2811723 | 2.82 | 13.57 | 1.02 | -0.50 |  |  |
|  | *qBR10_3* | 10 | Marker856739 | 2496414 | Marker881068 | 1530799 | 2.95 | 14.14 | 1.08 | -0.38 |  |  |
|  | *qBR11_1* | 11 | Marker2102110 | 5764052 | Marker2056824 | 4504984 | 2.65 | 12.81 | 0.91 | -1.07 | Inside | Branching 1-1 (Chen et al., 2007) |
|  | *qBR13_1* | 13 | Marker1045635 | 33388825 | Marker1094632 | 32571480 | 2.95 | 23.45 | 0.13 | -2.67 |  |  |
|  | *qBR14_1* | 14 | Marker1792639 | 38716712 | Marker1643198 | 44087851 | 4.68 | 35.73 | 1.71 | -1.38 |  |  |
|  | *qNode3_1* | 3 | Marker51677 | 34432502 | Marker85007 | 35368824 | 11.44 | 26.12 | 0.13 | -4.46 |  |  |
|  | *qNode3_2* | 3 | Marker34365 | 41212483 | Marker37993 | 44054643 | 8.82 | 14.82 | -0.17 | 2.97 |  |  |
|  | *qNode9_1* | 9 | Marker725322 | 1573416 | Marker656516 | 789646 | 4.28 | 4.67 | 0.02 | 2.94 |  |  |
|  | *qNode15_2* | 15 | Marker2256742 | 10683143 | Marker2248389 | 6350450 | 17.39 | 83.82 | 0.04 | -12.54 |  |  |
|  | *qNode20_1* | 20 | Marker1112314 | 2032772 | Marker1291840 | 23479729 | 7.42 | 11.07 | -1.53 | 0.04 |  |  |
|  | *qPod2_1* | 2 | Marker1894321 | 42679545 | Marker1907855 | 43898375 | 3.49 | 49.30 | 68.55 | -42.72 |  |  |

| Table S4 Identification of additional QTL by removing the effect of *E1* gene in Y133 population. | | | | | | |  |  |  |  |  |  |
| --- | --- | --- | --- | --- | --- | --- | --- | --- | --- | --- | --- | --- |
| Population | QTL | Chr | LeftMarker | Physical position (bp) | RightMarker | Physical position (bp) | LOD | PVE(%) | Add | Dom | Distance to known QTL or gene (kb) | QTL in SoyBase or known gene |
| Y133-*E1* | *qFT1_1* | 1 | Marker860296 | 49059102 | Marker889667 | 48966442 | 4.92 | 14.37 | -2.53 | -0.56 |  |  |
|  | *qFT6_2* | 6 | Marker1449694 | 46856851 | Marker1596115 | 45088674 | 7.36 | 29.52 | -4.08 | 0.58 | 762.59kb | First flower 1-2 (Keim et al., 1990) |
|  | *qFT7_2* | 7 | Marker1838814 | 42480669 | Marker1823729 | 43139642 | 3.70 | 4.88 | 1.76 | 1.38 |  |  |
|  | *qFT18_2* | 18 | Marker2566070 | 2491200 | Marker2566544 | 2374218 | 3.30 | 8.84 | 0.12 | 3.11 | 624.28kb | First flower 21-4 (Reinprecht et al., 2006) |
|  | *qFT20_2* | 20 | Marker1380631 | 43548635 | Marker1408825 | 47015159 | 10.50 | 58.84 | -0.17 | -9.06 |  |  |
|  | *qPH19_2* | 19 | Marker1629638 | 44862177 | Marker1666593 | 45184768 | 3.20 | 41.35 | 5.45 | 29.49 | 0.23kb | Plant height 10-4 (Orf et al., 1999) |
|  | *qPH19_3* | 19 | Marker1620683 | 40760282 | Marker1769282 | 40958504 | 2.98 | 42.27 | 14.61 | 1.26 | 578.02kb | Plant height 8-3 (Orf et al., 1999) |
|  | *qBR2_1* | 2 | Marker949955 | 11687058 | Marker1059871 | 11367075 | 3.06 | 14.01 | 0.61 | 2.11 |  |  |
|  | *qBR5_1* | 5 | Marker3267895 | 34369728 | Marker3340114 | 31545456 | 3.04 | 19.06 | -0.83 | -2.34 | Inside | Branching 4-1 (Yao et al., 2015) |
|  | *qBR6_1* | 6 | Marker1570324 | 17656443 | Marker1578129 | 16421211 | 4.72 | 45.32 | -2.29 | 1.18 | 1899.28kb | Branching 3-1 (Sayama et al., 2010) |
|  | *qBR7_1* | 7 | Marker1835020 | 37550684 | Marker1860076 | 3572434 | 3.26 | 27.27 | -1.80 | 0.30 |  |  |
|  | *qBR8_1* | 8 | Marker2713366 | 40908851 | Marker2800357 | 38375557 | 2.61 | 12.51 | -0.97 | 0.38 |  |  |
|  | *qNode6_2* | 6 | Marker1490011 | 9213156 | Marker1524454 | 8060722 | 2.85 | 25.30 | 0.53 | -3.05 |  |  |
|  | *qNode6_3* | 6 | Marker1523371 | 5747476 | Marker1535031 | 3027287 | 3.08 | 31.93 | 0.20 | -3.25 |  |  |
|  | *qNode8_1* | 8 | Marker2713366 | 40908851 | Marker2800357 | 38375557 | 2.90 | 41.84 | -1.81 | 1.21 |  |  |
|  | *qNode9_1* | 9 | Marker3118242 | 2275187 | Marker3120808 | 1116389 | 2.51 | 22.93 | -1.32 | 1.08 |  |  |
|  | *qNode20_1* | 20 | Marker1258177 | 37795328 | Marker1320117 | 40695692 | 2.67 | 24.37 | 0.34 | -2.71 |  |  |
|  | *qPod11_2* | 11 | Marker1183720 | 6021949 | Marker1210060 | 4627307 | 2.76 | 23.99 | 7.07 | 64.82 | 340kb | Pod number 3-1 (Sun et al., 2006) |
|  | *qPod15_2* | 15 | Marker476641 | 5187960 | Marker330620 | 4141915 | 2.56 | 22.82 | 22.47 | -45.67 |  |  |
|  | *qPod19_2* | 19 | Marker1635921 | 41294702 | Marker1620683 | 40760479 | 3.04 | 43.48 | 43.31 | -24.94 | 321.49kb | Pod number 9-3 (Kuroda et al., 2013) |
| Y133-*e1* | *qFT6_3* | 6 | Marker1550448 | 43529248 | Marker1596115 | 45088674 | 7.62 | 32.58 | -2.49 | -11.72 | 461.41kb | First flower 12-1 (Zhang et al., 2004) |
|  | *qFT6_4* | 6 | Marker1547068 | 11086775 | Marker1452469 | 10820805 | 3.08 | 4.52 | -2.30 | -3.35 |  |  |
|  | *qFT6_5* | 6 | Marker1523034 | 5949576 | Marker1536475 | 3149103 | 3.86 | 11.93 | 3.57 | -0.66 |  |  |
|  | *qFT16_1* | 16 | Marker2199160 | 175187 | Marker2153289 | 119447 | 7.36 | 34.73 | -0.60 | 12.36 |  |  |
|  | *qPH6_1* | 6 | Marker1434937 | 15504018 | Marker1558850 | 13723869 | 2.94 | 47.72 | -20.36 | -24.52 |  |  |
|  | *qPH8_1* | 8 | Marker2711396 | 17240120 | Marker2770106 | 15919776 | 2.55 | 52.93 | -1.06 | 45.74 |  |  |
|  | *qPH15_1* | 15 | Marker394633 | 14911800 | Marker480088 | 17330244 | 3.40 | 56.64 | 11.74 | -36.26 | 1257.82kb | Plant height 29-4 (Liu et al., 2011) |
|  | *qBR14_1* | 14 | Marker2886171 | 345481 | Marker2833216 | 452968 | 2.57 | 43.23 | -1.73 | -0.53 |  |  |
|  | *qBR15_1* | 15 | Marker481532 | 1825138 | Marker437611 | 1984973 | 2.84 | 46.43 | 0.65 | 2.66 |  |  |
|  | *qBR17_1* | 17 | Marker52634 | 11719926 | Marker98971 | 12110861 | 3.56 | 54.16 | -1.35 | -1.50 | Inside | Branching 3-4 (Sayama et al., 2010) |
|  | *qBR17_2* | 17 | Marker134981 | 37768211 | Marker100052 | 38188111 | 3.18 | 50.60 | -1.37 | -2.53 |  |  |
|  | *qNode4_1* | 4 | Marker649171 | 5294909 | Marker673149 | 1650768 | 3.04 | 7.65 | -1.26 | -1.69 |  |  |
|  | *qNode6_4* | 6 | Marker1587916 | 34230280 | Marker1561476 | 44179958 | 5.37 | 30.58 | 0.34 | -5.33 | 189.3kb | Node number 1-4 (Gai et al., 2007) |
|  | *qNode9_2* | 9 | Marker3125692 | 8507082 | Marker3083552 | 8701969 | 2.55 | 5.88 | -0.41 | 1.73 |  |  |
|  | *qNode10_1* | 10 | Marker2050793 | 51561569 | Marker2002012 | 49350327 | 2.69 | 6.12 | 0.36 | 2.45 |  |  |
|  | *qNode15_1* | 15 | Marker318422 | 9999755 | Marker394993 | 14823774 | 5.35 | 31.79 | 0.04 | -5.55 |  |  |
|  | *qNode16_2* | 16 | Marker2199160 | 175187 | Marker2153289 | 119447 | 3.40 | 8.34 | -1.28 | 0.00 |  |  |
|  | *qNode17_1* | 17 | Marker130433 | 7779356 | Marker135800 | 5852893 | 4.23 | 8.89 | -0.15 | 2.29 | Inside | Node number 7-1 (Li et al., 2009) |
|  | *qNode19_2* | 19 | Marker1695229 | 42565531 | Marker1722002 | 44921078 | 3.25 | 14.37 | 0.22 | 2.74 |  |  |
|  | *qPod11_2* | 11 | Marker1191778 | 4626607 | Marker1158449 | 4567661 | 2.82 | 52.77 | 20.64 | 56.72 |  |  |
|  | *qPod17_1* | 17 | Marker71932 | 4835831 | Marker57299 | 3850128 | 2.54 | 42.84 | -26.06 | -6.53 |  |  |

| Table S5 Identification of additional QTL by removing the effect of *E2* gene in Y159 population. | | | | | | |  |  |  |  |  |  |
| --- | --- | --- | --- | --- | --- | --- | --- | --- | --- | --- | --- | --- |
| Population | QTL | Chr | LeftMarker | Physical position (bp) | RightMarker | Physical position (bp) | LOD | PVE(%) | Add | Dom | Distance to known QTL or gene (kb) | QTL in SoyBase or known gene |
| Y159-*E2* | *qFT16_1* | 16 | Marker133751 | 1744685 | Marker4884 | 1469283 | 2.55 | 19.34 | 0.85 | 5.78 |  |  |
|  | *qFT18_1* | 18 | Marker2873468 | 8587965 | Marker2951053 | 7774568 | 4.00 | 60.20 | 4.25 | -1.77 | 742.96kb | First flower 9-2 (Reinprecht et al., 2006) |
|  | *qPH2_1* | 2 | Marker2073773 | 43210350 | Marker1997222 | 41732879 | 3.09 | 74.12 | 6.56 | 13.54 | 700.31kb | Plant height 26-9 (Sun et al., 2006) |
|  | *qPH4_1* | 4 | Marker2251035 | 41180835 | Marker2328095 | 13336223 | 2.62 | 45.61 | 10.64 | 16.83 |  |  |
|  | *qBR2_1* | 2 | Marker2084568 | 285567 | Marker2046426 | 1200000 | 3.85 | 58.91 | -2.34 | -3.49 |  |  |
|  | *qBR4_1* | 4 | Marker2211959 | 14076044 | Marker2180729 | 12902708 | 3.60 | 57.78 | -1.88 | -4.37 |  |  |
|  | *qBR10_1* | 10 | Marker2491207 | 46582574 | Marker2562044 | 48212365 | 3.73 | 64.74 | 0.46 | 4.42 |  |  |
|  | *qBR12_1* | 12 | Marker259797 | 39092894 | Marker190876 | 2635815 | 2.63 | 58.77 | -0.53 | 4.81 | Included | Branching 5-3 (Shim et al., 2018) |
|  | *qNode2_1* | 2 | Marker2004129 | 38592420 | Marker2057410 | 38154226 | 4.29 | 2.12 | -0.54 | -0.06 | 66.8kb | Node number 4-1 (Liu et al., 2011) |
|  | *qNode4_1* | 4 | Marker2263186 | 3853836 | Marker2194298 | 3567846 | 9.25 | 9.34 | 0.02 | -2.46 |  |  |
|  | *qNode9_1* | 9 | Marker1445830 | 49555447 | Marker1384932 | 48059714 | 8.28 | 7.48 | 0.01 | 1.93 |  |  |
|  | *qNode10_1* | 10 | Marker2581131 | 45301061 | Marker2607150 | 46746333 | 15.76 | 43.82 | 4.91 | -4.43 |  |  |
|  | *qPod12_1* | 12 | Marker160225 | 34446165 | Marker277796 | 37051858 | 2.74 | 55.29 | ##### | 78.28 |  |  |
